# Signaling Pathways in Inflammation and Its Resolution: New Insights and Therapeutic Challenges

**DOI:** 10.3390/ijms241311055

**Published:** 2023-07-04

**Authors:** Carla Cicala, Silvana Morello

**Affiliations:** 1 Department of Pharmacy, School of Medicine and Surgery, University of Naples Federico II, 80131 Naples, Italy; 2 Department of Pharmacy, University of Salerno, 84084 Fisciano, Italy; smorello@unisa.it

Tissue inflammation is a dynamic process that develops step by step, in response to an injury, to preserve tissue integrity. Resident cells are firstly involved, and once activated, they produce proinflammatory mediators and cytokines that, in turn, activate downstream proinflammatory signals. Blood cells are recruited into the inflammatory environment and activate a phagocytic mechanism to eliminate debris and microorganisms.

Chronic tissue inflammation may represent a risk factor for many diseases, such as autoimmune diseases and malignancies. In the past, therapeutic strategies to fight tissue inflammation have mainly been based on the inhibition of proinflammatory mediators and signaling. The resolution of inflammation is an active process that enables inflammation to switch off, protecting tissue from persistent injury and restoring tissue homeostasis. Like inflammation, resolution involves several mediators and signaling pathways that control immune cell functions. A host’s failure to resolve inflammation may be caused by a pathological mechanism driving tissue towards chronic inflammatory conditions. In recent years, the research in this field has been working towards clarifying these pro-resolving pathways and signaling pathways that may be targets for therapeutic intervention [1,2].

The Special Issue entitled *“Signaling Pathways in Inflammation and Its Resolution: New Insights and Therapeutic Challenges”* includes five original papers and two reviews in which international experts present new information about novel molecular targets for possible therapeutic strategies to treat inflammatory disease.

In line with recent evidence supporting integrative omics as an important tool for the prognosis and diagnosis of different diseases, Hernandez-Baixauli and coworkers [3] highlighted the need for animal models of chronic inflammation, which are useful in the study of metabolomes of different body fluids. They described a rat model of chronic inflammation induced by the intermittent injection of bacterial lipopolysaccharide (LPS); they characterized the model by an increase in plasma of inflammatory biomarkers, such as monocyte chemoattractant protein-1 (MCP-1) interleukin-6 (IL-6), tumor necrosis factor-α (TNFα), and prostaglandin E_2_ (PGE_2_). At the same time, by analyzing the plasma metabolome, they found alterations in lipid metabolism, with a particular increase in fatty acid and cholesterol metabolic pathways, glycerophospholipid metabolism, and sphingolipid metabolism, and a reduction in tricarboxylic acid (TCA) metabolism.

Another important issue was addressed by Lee and co-workers [4]. Following experiments performed on mice expressing human-sex-hormone-binding globulin (SHBG) and on their wild-type (WT) counterparts, they found that the dietary intake of 17α-ethinyl estradiol (EE2) promotes the progression of hepatocellular carcinoma (HCC) in mice expressing SHBG protein but not in WT mice. The authors demonstrated that this effect is likely dependent upon the ability of EE2 to bind SHBG and to increase, in this way, the bioavailability of androgen. The authors found that the effect of EE2 in SHBG-expressing mice is due to increased inflammation, associated with increased androgen-dependent proliferation in the liver. On the bases of evidence that prostate cancer patients can be exposed to liver cancer following estrogen therapy, the authors concluded that those men treated with EE2 who have high SHBG levels may have an increased risk of developing HCC.

Several eye diseases are associated with ischemia reperfusion injury (IRI). H_2_S is the most recently identified endogenous gas transmitter; it regulates several cell functions, and it is beneficial in several inflammatory conditions and tissue injury. Scheid and coworkers [5] highlighted the important role of H_2_S in neuronal injury following the ischemia–reperfusion of the retina in rats. Based on the observation that low doses of H_2_S may offer neuroprotection, in contrast to high H_2_S concentrations, which may be toxic [6,7], they investigated the effect of H_2_S inhalation in a rat model of retinal ischemia–reperfusion injury. The results obtained in this study demonstrated that H_2_S inhalation is beneficial in the ischemia–reperfusion injury of neuronal cells, in a dose- and time-dependent manner, especially at 80 ppm given 1.5 h after reperfusion. The authors showed that a neuroprotective effect is achieved via the inhibition of NF-kB activation, the reduced expression of the pro-inflammatory cytokines IL-1ß and TNF-α, the decreased expression of the pro-apoptotic protein Bax, and the increased expression of the anti-apoptotic Bcl-2.

Antonioli and coworkers [8] addressed the importance of metabolic changes in the gut epithelial lining in inflammatory bowel diseases; in particular, they highlighted the finding that the reduced expression and activity of AMP-activated protein kinase (AMPK) has been observed in several immune inflammatory diseases, including intestinal bowel diseases (IBD). It is known that AMPK is a kinase important in driving cell polarization towards an immunoregulatory phenotype [9]. Therefore, they evaluated the efficacy of a novel direct AMPK activator in an experimental model of colitis in rats. They compared the effect of the AMPK activator, FA-5, synthesized in their laboratories, with the commercially available acadasine, an AMPK activator that requires bioactivation, and with dexamethasone. By performing in vitro experiments, they demonstrated that FA-5 activates AMPK and SIRT1, in line with evidence that the AMPK/SIRT-1 enzyme axis has a crucial role in counteracting immune-inflammatory processes [10]. They also found that the pharmacological activation of AMPK, via FA5 incubation, elicits a shift in cellular metabolism from glycolytic metabolism, known to be typically increased in inflammatory cells [11], to the β-oxidation of fatty acids and oxidative metabolism, typically utilized to generate energy by Treg [12]. In vivo, in a model of 2,4-dinitrobenzenesulfonic acid (DNBS)-induced colitis in rats, the authors observed a reduction in TNF and an increase in the anti-inflammatory cytokine IL-10 in the colonic tissues from animals with colitis treated with FA5 or acadesine. They highlighted the importance of AMPK as a molecular target for new therapeutic strategies in chronic intestinal inflammation.

Acute lung injury (ALI) is a clinical disorder with a high mortality rate; together with acute respiratory distress syndrome (ARDS), it is the main cause of respiratory failure associated with high morbidity and mortality rates [13]. Palmitoylethanolamide (PEA) is an endogenous lipid with anti-inflammatory and analgesic activity, and in a murine model of allergic airway inflammation, it was shown to be able to inhibit mast cell degranulation. Peritore and coworkers [14] evaluated the effect of the oral administration of ultramicronized palmitoylethanolamide (um-PEA) in a model of LPS-induced ALI in mice. They found reduced inflammatory markers and cell accumulation in the lungs of mice treated with um-PEA. Furthermore, they showed that treatment with um-PEA is able to stabilize lung mast cell degranulation and to reduce chymase and tryptase activity after LPS-induced ALI. They demonstrated that um-PEA inhibits nuclear factor kB (NF-kB) pathway activation and downstream signaling in lung tissue.

CD83 is a molecule expressed in a variety of cells. It is present as a membrane-bound isoform (mCD83) highly expressed on activated immune cells, such as dendritic cells (DCs) and B cells, and it is also released as a soluble isoform (sCD83) [15,16]. High serum levels of CD83 have been found in chronic inflammatory diseases. In their review, Peckert Maier and coworkers collected evidence of the role of CD83 as an important molecule in the resolution of inflammation [17]. Several experimental data highlight an impaired immune cell function in CD83^−/−^ mice. There are data demonstrating that the administration of sCD83 in animal models of inflammation and transplantation leads to the resolution of inflammation and enhances the allograft survival rate. They outlined the function of mCD83 in inflammation as a regulator of DC and Treg and the sCD83 isoform as important in resolving inflammation in autoimmune diseases and in inducing tolerance in transplantation.

The review by Korbecki and coworkers [18] discussed the importance of making a comparison between chronic and cycling hypoxia within the tumor microenvironment. Although both activate hypoxia-inducible factor-1 (HIF-1) and NF-kB and alter the expression of similar genes, in cycling hypoxia, the activation of HIF-1 appears to be longer; furthermore, the expression level of the HIF-1α protein is increased with successive hypoxia cycles. The authors outlined the importance of considering that cyclic hypoxia occurs in the most considerable part of a tumor, while chronic hypoxia affects between 25 and 52% of the tumor area, and the need for in vitro experiments that reproduce cycling rather than chronic hypoxia.

Overall, these papers provide new insights into the mechanisms of activation of some inflammatory pathways and signaling pathways that could be helpful in the identification of potential therapeutic targets to control inflammation.
